# The uterine secretome initiates growth of gynecologic tissues in ectopic locations: re-evaluating the evidence

**DOI:** 10.3389/pore.2026.1612281

**Published:** 2026-03-10

**Authors:** Jan Sunde, K. A. Pennington

**Affiliations:** 1 Division of Gynecologic Oncology, Department of Obstetrics and Gynecology, Baylor College of Medicine, Houston, TX, United States; 2 Basic Sciences and Perinatology Research Laboratories, Department of Obstetrics and Gynecology, Baylor College of Medicine, Houston, TX, United States

**Keywords:** endosalpingiosis, endometriosis, implantation, ovarian cancer origin, uterine secretome, Mullerian agenesis, coelomic metaplasia, secondary Mullerian system

## Abstract

The origin of ectopic gynecologic lesions has been debated since 1927, when Sampson first proposed retrograde menstruation as the underlying cause of endometriosis. Reproduction in mammals is an unusually permissive process, enabling the implantation of tissue genetically distinct from the mother in which leukemia inhibitory factor (LIF) is known to be a pleiotropic master transcription factor affecting multiple gene pathways such as adhesion and immune tolerance. Herein we review the *uterine secretome theory*, and how the initial step in ectopic lesion development is implantation. The uterine secretome, which typically cycles every 28–35 days to prepare the endometrium for potential embryo implantation and does so for decades, can be hijacked by free floating cells to implant ectopically when pregnancy does not occur. This review will focus on this emerging theory and its ability to reconcile longstanding gaps in our understanding of both benign and malignant ectopic lesion initiation.

## The origin of benign and malignant ectopic gynecological lesions: a century of debate

The origin of ectopic gynecologic lesions has been debated since 1927, when Sampson first proposed retrograde menstruation as the underlying cause of endometriosis, challenging the prevailing view that lesions arose from ovarian cysts [1]. Reproduction in mammals is an unusually permissive process, enabling the implantation of tissue genetically distinct from the mother in which leukemia inhibitory factor (LIF) is known to be a pleiotropic master transcription factor affecting multiple gene pathways such as adhesion and immune tolerance [2]. Until recently, however, this unique biological feature had not been considered as a mechanism contributing to ectopic lesion formation.

**FIGURE 1:**
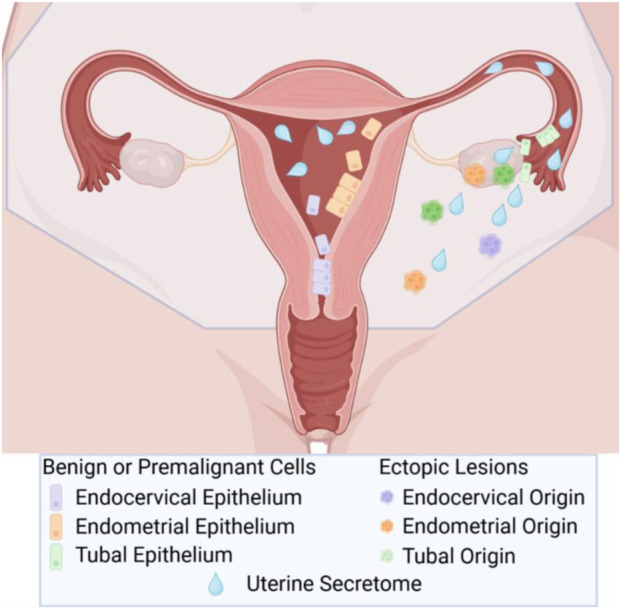
Overview of secretome hypothesis. Through retrograde menstruation, benign and premalignant endocervical, endometrial, and tubal epithelial cells migrate into the peritoneum. Factors produced by the uterus essential for embryo implantation, termed the uterine secretome, also flow into the peritoneum and promote implantation of these cells and development into benign and malignant lesions. Created in BioRender. Pennington, K. https://BioRender.com/ vtkyaze.

A recent publication introduced the *uterine secretome theory*, which builds upon the retrograde menstruation hypothesis and, using a mouse model, provides evidence that the initial step in ectopic lesion development is implantation driven by the uterine secretome [3]. The uterine secretome, which typically cycles every 28–35 days to prepare the endometrium for potential embryo implantation and does so for decades, can be hijacked by free floating cells to implant ectopically when pregnancy does not occur (Figure 1). This review will focus on this emerging theory and its ability to reconcile longstanding gaps in our understanding of both benign and malignant ectopic lesion initiation. Contending hypotheses, including coelomic transformation, embryonic rests, and metaplasia, have each retained advocates [4]. Notably, some early proponents of these theories recognize a potential role for the uterine tubes. Sampson himself postulated in the 1920s that factors originating from the uterine tubes, which he termed “digestive ferment,” might contribute to endometriotic adhesions [1]. Similarly, Novak, a supporter of the coelomic metaplasia theory, proposed in 1932 that “The only other explanation would be that an adventitious factor is added by some substance emanating from the ends of the tube”.

The cellular origination of ovarian cancer has also been controversial, with theories including ovarian surface cell metaplasia, uterine tubal cells, and an origin in the secondary Mullerian system [5].

### Ectopic benign and premalignant lesions

Endosalpingiosis (ES), defined as the ectopic growth of uterine tubal tissue, has attracted renewed attention since a 2016 retrospective chart review found ES associated with gynecological malignancy in 42% (354/838) of cases, with an overall prevalence of ∼1.5% in 60,000 gynecologic specimens [6]. However, a subsequent study using more intensive pathologic evaluation, particularly the Sectioning and Extensively Examining the FIMbriated end (SEE-FIM) protocol, demonstrated much higher rates of benign ectopic lesions. In women aged 31–50 undergoing gynecologic surgery, prevalence was 37% for ES, 32% for endometriosis (EM), 47% for paratubal cysts (PTC), and 29% for Walthard’s nests (WN, ectopic urothelial cell growths). After menopause, ES prevalence rose sharply to 66%, whereas EM declined dramatically to 5%. Overall, ectopic lesions were nearly ubiquitous after menopause, with 89% of specimens demonstrating at least one lesion type, and multiple lesion types frequently co-occurring within the same patient [7]. These findings reveal a very robust process that stands in stark contrast to the 1.5% prevalence previously reported and underscore how more extensive sampling methods reveal the extent of ectopic lesions. Importantly, they also highlight the potential for sampling bias in retrospective studies linking ES, EM, WN, or PTC to malignancy, since the patients undergoing cancer surgery are more likely be postmenopausal and to receive more thorough pathologic assessment than those with benign lesions [8–10]. Of note, the 32% prevalence of EM reported in gynecologic specimens with ovaries present in women age 31–50 over 1 year [7] is also substantially higher than the 5%–10% typically cited in the literature, which is largely based on women presenting for endometriosis treatment.

Recent clinical evidence increasingly supports the role of the uterine secretome in driving implantation of pre-malignant lesions. A large 2022 study reported that women undergoing risk-reducing salpingo-oophorectomy (RRSO) with serous tubal intraepithelial carcinomas (STIC) had markedly elevated risk of developing primary peritoneal serous carcinoma (PPSC) compared to women with normal Fallopian tube epithelium [11]. The study observed PPSC rates of 10% at 5 years and 25% at 10 years, with a hazard ratio of 33.9. The most plausible explanation is that pre-malignant serous lesions had implanted in the peritoneum prior to RRSO and subsequently underwent malignant transformation.

The secretome theory also provides a unifying explanation for long-standing epidemiologic findings that bilateral tubal ligation and hysterectomy reduce ovarian cancer risk [12–15], including for the serous subtype [16], despite the fimbriae left *in-situ*. Tubal ligation has also been associated with reduced risk of uterine serous and endometrial carcinoma [17, 18], a fact readily explained by the secretome theory. Furthermore, evidence that earlier RRSO confers greater cancer risk protection [11, 19] and that opportunistic salpingectomy (OS) reduces the risk of multiple ovarian epithelial cancer subtypes with a HR of 0.2 [20], is also consistent with this framework.

### Mouse model of ectopic lesion implantation

A mouse model using tdTomato-labeled minced gynecologic tissues injected into a wild type C57/B6 mice demonstrated that ectopic lesion formation was enhanced in the presence of secretory-phase endometrium, driven by cyclic estrogen and progesterone. Lesion implantation was further promoted by leukemia inhibitory factor (LIF), a cytokine essential for mouse embryo implantation [3]. Although Sampson proposed retrograde menstruation as the mechanism for endometriosis in the 1920s, the parallel between the endometrium’s capacity for embryo implantation and its potential to facilitate ectopic lesion implantation had not been previously explored. To further investigate this process in the context of ovarian cancer initiation, we have developed a fluorescent PTEN/DICER double knockout mouse model, which exhibits significantly increased ectopic lesion implantation in response to LIF (unpublished data).

## Genetic and epigenetic contributions to ectopic lesion development

Recent research has added complexity to theories on ectopic lesion origin, implicating genetic and epigenetic changes and inflammatory signaling in the establishment and persistence of EM. Gene expression studies in endometriosis [21], ovarian cancer metastasis [22], and embryo implantation [23, 24], consistently report altered gene expression of LIF.

Lists of differentially expressed genes (DEGs) and miRNAs have been generated while studying the endometrium [25–32] which identify genes and miRNAs that are also reported to have roles in implantation, endometriosis, and ovarian cancer. These lists are analogous to “ingredient lists” without baking instructions, and provide important insight but require further functional interpretation and resolution of conflicting findings to better understand the role of the uterine secretome in initiation of implantation, EM, and ovarian cancer.

One comprehensive list of differentially expressed genes and miRNAs which includes both uterine fluid and endometrial biopsies sampled sequentially through the menstrual cycle was published in 2025 [25]. Forty-three miRNAs were differentially expressed between the proliferative and early secretory phase, with 5 of the miRNAs altered in the opposite direction between EVs and the endometrium, potentially signaling the blastocyst [25] and playing a role in EM and ovarian cancer initiation (Table 1).

**TABLE 1:** Differentially expressed miRNAs in EVs with opposite expression compared to endometrial biopsy during early secretory phase versus proliferative phase [25].

miRNA	Ovarian cancer	EM	Implantation
miR-200c-3p	[33]	[34]	[35]
miR-449a	[36]	[37]	[38]
miR-10a-5p	[39, 40]	​	[41]
miR-200a-5p	[42]	​	[43]
miR-10a-3p	[44]	​	​

Seven of the 9 differentially expressed miRNAs upregulated in the mid- versus early- secretory phase have also been independently associated with EM and ovarian cancer (Table 2).

**TABLE 2:** miRNAs expressed between early secretory and mid secretory phase [25].

miRNA	Ovarian cancer	EM	Implantation
miR-31-5p	[45]	[46]	[47]
miR-34c-3p	​	[48]	[49]
miR-200b-5p	[50]	​	[51, 52]
miR-200b-3p	[50]	[53]	[51, 52]
miR-141-3p	[50]	[54]	[52, 55]
miR-200a-3p	[50, 56]	[56, 57]	[52]
miR-429	[50]	[58]	[52]
miR-30d-5p	[59]	[60]	[27, 61]
miR-885-5p	[62]	[63]	[64]

Another report of the top 20 secretory-phase DEGs has nearly all DEGs implicated in implantation, EM and ovarian cancer (Table 3), suggesting shared molecular drivers.

**TABLE 3:** Up-regulated differentially expressed genes in the secretory phase [31].

Gene symbol	Gene name	Ovarian cancer	EM	Implantation
GPX3	Glutathione peroxidase 3 (plasma)	[65]	[66]	[67–69]
PAEP	Progestagen-associated endometrial protein	[70]	[71, 72]	[73–75]
COMP	Cartilage oligomeric matrix protein	[76]	​	[77]
SLC1A1	Solute carrier family 1 (neuronal/epithelial high affinity glutamate transporter, system Xagl, member 1	​	​	[69]
LIF	Leukemia inhibitory factor (cholinergic differentiation factor)	[78, 79]	[80, 81]	[73, 82]
TCN1	Transcobalamin I (vitamin B12-binding protein, R binder famly)	​	​	​
CXCL14	Chemokine (C-X-C motif) ligand 14	[83, 84]	[85]	[26, 75]
C4BPA	Complement component 4 binding protein, alpha	[86, 87]	​	[88]
TSPAN8	Tetraspanin 8	[89–91]	​	​
LAMB3	Laminin, beta 3, transcript variant 2	[92, 93]	​	[94]
MAOA	Monoamine oxidase A, nuclear gene encoding mitochondrial protein	[95]	​	[96]
SOD2	Superoxide dismutase 2, mitochondrial, nuclear gene encoding mitochondrial protein, transcript variant 2	[97, 98]	​	[82]
GADD45A	Growth arrest and DNA damage inducible, alpha	[99]	​	[82]
MUC16	Mucin 16, cell surface associated	[100]	​	[101]
THBD	Thrombomodulin	[102]	​	[103]
NNMT	Nicotinamide N-methyltransferase	[104]	​	[67]
DPP4	Dipeptidylpeptidase 4 (CD26, adenosine deaminase complexing protein 2)	[105]	​	[106]
SCGB2A2	Secretoglobin, family 2A, member 2	[107]	​	[108]
S100P	S100 calcium-binding protein P	[109]	​	[110]

As in baking, timing is also critical in the implantation process. For example, miR141-3p is decreased in EM compared to eutopic endometrium [54], yet is increased in the mid-secretory compared to early secretory phase of the cycle [25]. Such context-dependent regulation illustrates the dynamic role of miRNAs and genes depending on the phase of the menstrual cycle. Additional complexity arises from the role of circular miRNAs [111] and long non-coding miRNA [112], underscoring how much remains to be clarified about gene-miRNA interactions in implantation and ectopic lesion development.

Endometriotic lesions, despite their benign histology, have been found to harbor cancer-driver mutations [113]. EM patients have been shown to have elevated LIF in the peritoneal fluid [114, 115]. Recent research proposing genetic and epigenetic changes as a cause of EM, including mutations in KRAS and ARID1A genes, in eutopic and ectopic endometrium [116, 117], further support the secretome theory. These genes regulate processes driven by the uterine secretome, including implantation, cell invasion and migration, which are essential for blastocyst implantation, raising the possibility that women carrying these mutations are predisposed to develop EM, perhaps explaining why only 32% of women aged 31–50 undergoing gynecologic surgery with ovaries removed were found to have lesions [118], in contrast to the 50%–90% of women [119] believed to experience retrograde menstruation.

The molecular drivers of lesion initiation remain poorly defined, but accumulating evidence suggests that dysregulated miRNAs involved in proliferation and invasion will likely be found to be critical for the implantation pathway initiated by the uterine secretome. For example, miRNAs detected in both EM and uterine secretory exosomes, such as miR-302a and let-7b-5p, represent promising epigenetic regulators requiring further investigation [120, 121]. Additional data supporting the secretome theory include the association of EM derived exosomes with increased invasion and migration [122], and the persistence of LIF expression into the menstrual phase [123]. The peritoneal cavity also presents a distinct environment compared to the endometrial cavity, with multiple reports describing immune responses with ectopic growth [124] accompanied by altered miRNA expression patterns [53, 57, 125]. LIF activates the JAK-STAT signaling pathway, which is also altered in ovarian metastasis [79], implicating LIF in ovarian cancer. Further study of overlapping gene and miRNA dysregulation across implantation, EM, and ovarian cancer may clarify shared molecular drivers of benign, pre-malignant, and malignant lesion initiation.

Reports show that borderline and low grade carcinomas, as well as associated benign ES lesions, can share identical genetic mutations [126], suggesting that some benign lesions may serve as potential precursors arising in the tube. However, other data challenge this interpretation. For example, one study found that 57% of patients had no identifiable lesions or genetic mutations noted in the tubes [127], raising the possibility that a spectrum of cells, some appearing histologically normal yet harboring early genetic mutations, may shed and subsequently implant ectopically.

While several studies report shared genetic mutations between benign and malignant lesions, additional reports describe associations between benign ectopic lesions (including EM and ES) with malignant lesions [6, 128–131]. Nevertheless, these associations may largely reflect sampling bias rather than true causality, given the near-ubiquitous presence of benign ectopic lesions in the general population.

## Fluid and cellular movement fits the secretome theory

Retrograde menstruation occurs in up to 90% of women [119], coinciding with myometrial contractions directed toward the cervix [132]. Endometrial cells have been identified in the peritoneal cavity in ∼50% of patients during both follicular and early secretory phases [133], and endosalpingial cells were reported in nearly all patients in one series of 38 patients [134]. Uterine contractility driving fluid toward the tubal ostia has been observed during the follicular and early secretory phase of the cycle [132]. Murine studies demonstrate similar mechanisms, an India ink bolus migrated toward the ovaries and into the ovarian bursa due to oviductal peristalsis, despite ciliary movement that should direct flow oppositely, with active fluid secretion occurring along the full length of the tube [135].

*In vivo* measurement following bilateral tubal ligation estimated mean oviductal fluid production of 35 mL per cycle (19 mL mid-cycle, 16 mL in the secretory phase) [136]. This is likely an underestimation given exclusion of the fimbrial portion. Uterine fluid production averages up to 30/mL day in the early secretory phase, decreasing to <10 mL/day, totaling ∼250 mL per cycle [137]. Hysterosalpingoscintigraphy with 99m-labelled macroaggregates demonstrated rapid vaginal-to-uterine transport, with particles carried toward the cornua and tubes in up to 79% of patients during the follicular and early secretory phases, often ipsilateral to the dominant follicle; 6% showed peritoneal spillage [138]. Ovarian follicular rupture releases ∼4 mL of fluid [139], and peritoneal fluid peaks mid-cycle at ∼23 mL, falling to ∼6 mL in the late secretory phase [140]. By contrast, men and postmenopausal women have only 2–3 mL of peritoneal fluid [139]. Notably, ninety percent of differentially expressed miRNAs in uterine EVs are also present in oviduct EVs [141], consistent with uterine EV transfer. Together, these findings support the plausibility of ectopic lesion implantation under the influence of the uterine secretome.

### Hormonal effects on ectopic lesions

The uterine secretome theory provides a framework for understanding the hormonal influences on initiation of benign ectopic lesions including ES, EM, PTCs and WN that typically emerge with the onset of hormonal cycling [142, 143, 144], increase with age, and become nearly ubiquitous after menopause [118], consistent with cumulative uterine cycles releasing secretory factors that promote lesion initiation and growth.

This theory also explains epidemiologic observations. For example, the decrease prevalence of OICs with prolonged oral contraceptive (OCP) use [145] and the decreased ovarian cancer risk associated with progesterone IUD use [146, 147] can be interpreted as consequences of suppressed uterine secretome activity [145]. While OCPs have traditionally been thought to decrease ovarian cancer risk by decreasing ovarian cellular metaplasia [148], an alternate explanation is that OCPs block the uterine secretome signaling required for lesion initiation. Similarly, suppression of EM symptoms by progesterone IUDs [149] or oral hormones [150], may result not only from direct hormonal effects but also from inhibition of new lesion formation by secretome suppression. Finally, the distant spread of EM and ES lesions to sites such as the lung [151] can also be accounted for by this mechanism, as extracellular vesicles and endometrial stromal cells have been found in the bloodstream of patients with EM [152, 153].

### Re-evaluating the evidence: unexplained data

Several long-puzzling findings can be explained by the role of the uterine secretome in ectopic lesion development. If alternate ovarian epithelial cancer origin theories were correct, tubal ligation and hysterectomy would increase ovarian cancer risk by allowing more ovulations after discontinuation of hormonal contraception. Instead, multiple studies report a decreased risk [13, 16, 154]. Precancerous lesions in the uterine tube have been reported in cases of uterine serous carcinoma [155], suggesting that tubal cells may implant ectopically in the endometrial cavity. Supporting this idea, ectopic ciliated cells within the uterus cluster near the tubal ostia [155], consistent with a tubal origin of the precursor cells of uterine serous carcinoma.

The secretome theory also clarifies why women with Mullerian agenesis (MA), who lack a uterus, rarely develop EM [156], or epithelial ovarian carcinoma [157]. Given that these women cycle from menarche to menopause without the risk-reducing effects of pregnancy [158] or breastfeeding [159], one would predict an increased ovarian cancer risk. Women with MA which has a prevalence of approximately 1/5000 [160] are subject to “incessant ovulation”, ovulating from menarche to menopause, and should have a higher risk of ovarian cancer than the expected 1.1% lifetime incidence reported in the US general population,^1^ yet there are less than 20 cases reported worldwide, consistent with the lack of cycling uterine secretome. Up to 10,000 cases or more would be expected worldwide in these women who have normal appearing distal tubes and ovaries in 90% of patients [161]. All the epithelial lesions have been reported to be serous or poorly differentiated ovarian cancers [157], likely arising in the fimbrial remnant of the uterine tube that is present. Similarly, EM is not expected in MA, and a review of EM occurring in MA women reported functional endometrium in nearly all cases, with questionable findings in a small number of cases [156], providing a potential source of uterine secretome factors driving lesion development in these women.

### Re-evaluating the evidence: competing theories of ectopic lesion development

Traditional theories, including Sampson’s retrograde menstruation hypothesis [27], embryonic rests [5], secondary Mullerian system [162], the coelomic metaplasia theory (Meyer, 1924 in German) leading to ectopic growths, and incessant ovulation with ovarian epithelial metaplasia [163] and the “precursor escape” proposed by Piek [164] as a source of ovarian cancer have not considered the uterine secretome’s role in the attachment and implantation process. Nor do they explain the reported 89% prevalence of benign epithelial ectopic lesions in post-menopausal patients [118]. The uterine secretome theory integrates and expands on retrograde flow and precursor escape models by identifying the initial implantation step mediated by uterine secretome factors. It explains the age-related increased in ectopic growths, as more secretory cycles lead to more lesions, with the notable exception of hormone driven cyclic EM, which declines after menopause [118]. Distant ectopic growths, presumably seeded by endometrial cells [152] and EVs [153] detected in the bloodstream, can also be explained by secretome-driven remote ectopic implantation.

Another variant of the theory of metaplasia after incessant ovulation [163] that has fallen out of favor is that follicular fluid driven by incessant ovulation may have a direct effect on fallopian tube epithelium [165]. Increased reactive oxidative stress (ROS) markers in patients undergoing IVF has been demonstrated in serum in humans undergoing IVF [166] as well as in follicular fluid (FF) in approximately 50% of IVF patients which notably had carcinogenic effects on FTE only in the presence of elevated ROS markers [167]. Proteomic analysis of ovarian stimulation vs. natural cycle IVF have reported alterations in multiple gene pathways, including free radical scavenging [168], suggesting that the effect of FF on carcinogenesis may not occur outside the construct of a laboratory study since only FF with elevated ROS affected FTE growth.

It has been demonstrated that superovulation of mice dramatically affects ovarian gene expression, as well as the endometrial gene expression and adversely affects implantation and fertility [169]. There is increased estrogen and decreased progesterone levels and dramatically decreased uterine LIF [169], potentially counteracting any stimulatory follicular fluid effect on the FTE. Another study found that Fallopian tube epithelium (FTE) underwent ROS associated changes when exposed to follicular fluid, with a lesser effect noted with peritoneal fluid from the late luteal phase (with no collection of fluid near mid-cycle to prevent an adverse effect on possible pregnancy) but not the follicular phase [170], so it may be that early to mid-secretory peritoneal or uterine fluid may have a greater effect on FTE. Controversy persists regarding the possibility of assisted reproduction affecting the risk of ovarian cancer [171], making it unlikely that the follicular fluid plays a significant role. Subsequent research has been published where excision of the tubal/ovarian bursa in mice led to increased peritoneal seeding after intraperitoneal injection of FTE cells with an attribution to the possible effects of the FF [172], but did not consider more copious fluid sources, i.e., the tube, uterus, or peritoneal fluid, nor the potential for rapid dilution of follicular fluid in the peritoneal cavity [167].

The focus of “incessant” cycling should be the endometrium and its secretome [3], rather than the ovary. The cycle is very sensitive to disruption in the lab and in humans, so data must be evaluated to determine whether the reported effects can be expected to occur during normal ovulatory/menstrual cycles and which hypothesis the data supports. The theories of incessant ovulation [163] or incessant release of ovarian follicular fluid [165] cannot explain this unexplained data, yet the uterine secretome theory [3] which entails the lack of “incessant” uterine secretome release does.

### Re-evaluating the evidence: caveats in interpreting menstrual cycle research

The menstrual cycle is often described as an endocrine symphony, with distinct phases leading to ovulation followed by embryonic implantation, driven by dynamic gene changes in the uterine fluid and endometrium throughout the cycle [25]. Just as in embryonic implantation, ectopic lesion implantation and subsequent growth are likely influenced by different hormonal and molecular cues. For example, co-culture of oviductal cells with endometrial cells increases LIF and avβ3 expression [173], a finding that would not be noted if studied separately. A study of LIF in uterine flushings from women with EM reported a 30%–40% difference (25 pg/mL vs. 36 pg/mL) in LIF from uterine flushings in a small number of EM patients, a finding that is not statistically significant [81], but is supported in other research of LIF and EM [114].

### Re-evaluating the evidence: intriguing data from mouse models

Animal models of EM have been questioned because most species do not menstruate, but mouse models remain valuable due to their short, hormonally similar ovulatory cycles and low cost [174]. Mouse models of ovarian inclusion cysts (OIC) mimic human findings, such as increases in incidence with age [175–177]. However, experimental designs often overwhelm the hormonal milieu, which can obscure physiological processes. Transgenic studies have identified genes implicated in OIC pathogenesis. For example, FOXA2 loss decreased EM lesion development and proposed prolonged treatment with LIF as a possible treatment strategy [178]. Timing is critical, in a pancreatic cancer model, LIF expression rose during the first 4 weeks of lesion implantation but then declined after establishment [179]. And in some settings, overwhelming the hormonal milieu can be an effective treatment strategy, as demonstrated by the availability of both GnRH agonists and antagonists for EM treatment [180]. Disadvantages of the mouse model of EM and ES that are important to mitigate include the lack of spontaneous menstruation, and the location of the oviduct and ovary within a bursa. A tdTomato model evaluated both menstrual and secretory endometrium, utilized a wild type recipient to mitigate off target effects, and injected minced tissue directly into the peritoneal cavity to avoid some of these potential pitfalls [3].

Multiple studies in mice have excised tubes or ovaries in an attempt to determine which might be the primary source of ovarian cancer, with both tubes and ovaries reported to be the origin [3, 181, 182], but careful attention must be paid to the timing of the procedure. A 2025 example is a study claiming that ovarian cancer may arise in ES, where the oviducts were excised at 8 weeks, 3–4 weeks after mice are capable of ovulating [183] providing multiple cycles every 4–5 days for cells to implant ectopically. Another report argued that the presence of a small number of OIC lesions in mice after 4 weeks of life supports the metaplasia theory [184]. However, these mice had tamoxifen stimulation at an age when mice are hormonally primed to cycle [185], suggesting the lesions may have arisen in response to uterine secretome activity. Importantly, a later study found no OICs were identified in 100 ovaries prior to initiation of ovulation (∼35 days of age) [3], underscoring the critical influence of cycle stage on study design.

Knockout models further support the secretome theory. In the DICER/PTEN double-knockout model, which develops tumors resembling serous carcinoma, ovariectomy at 6–11 weeks prevented cancer spread beyond the oviducts [186], likely due to lack of the uterine secretome and blocking access to the peritoneal cavity via the ovarian bursa. In the same model, early treatment with mifipristone largely prevented cancer development, whereas progesterone supplementation promoted dissemination with 66% of mice developing disease after 1 week of progesterone exposure, and 100% after 3 weeks [187]. These results suggest that progesterone-driven uterine secretome activity initiates ectopic lesion development, while sustained exposure promotes progression, paralleling progesterone’s role in the maintenance of pregnancy.

In a PTEN/DICER/TP53 triple-knockout model, ovarian epithelial metaplasia was proposed as a possible origin of ovarian cancer after oviduct excision at 2 months of age [182]. However, premalignant spread prior to excision was not considered. A subsequent study reported cancer as early as 2 months [188] again consistent with spread occurring prior to oviduct removal, given that mice begin ovulation by 5 weeks of age. Together, these findings highlight the need for careful temporal analysis of lesion initiation in these models.

### Re-evaluating the evidence: fitting theories with the data

Theories of lesion origin must be judged by how well they fit observed data. Evidence that ectopic gynecologic lesions are hormonally driven is consistent with the absence of prepubertal OIC lesions in mice [3]. ES has been reported as being “pre-pubertal” in 4 week old mice, but only after tamoxifen treatment [184]. Neonatal and premenarche cysts [144, 189] may reflect active cellular growth with cellular shedding into the peritoneal cavity [133, 190] under maternal or early premenarchal hormone stimulation. Supporting this, a mouse model demonstrated that detached EM & ES cells attach to surrounding tissue even in the absence of hormonal or secretome stimulation [3], providing a plausible mechanism for neonatal uterine bleeding as a source for early EM [191].

Sampson first observed in 1921 that EM declines sharply after menopause [192], a finding confirmed a century later using the See-FIM protocol on >500 gynecologic specimens [118]. Rare Mullerian lesions in males purportedly support metaplasia as a cause of ovarian cancer [193, 194], but the testis appendices, a Mullerian remnant found in 75% of males [195], offer a plausible source. The embryologic remnant theory also does not explain why benign ectopic growths, reported in 89% of post-menopausal women [118], are essentially absent in men. By contrast, the uterine secretome theory explains both the scarcity of ectopic lesions in MA patients [156], where no secretome is present, and the consistent finding that MA patients with EM harbor a uterine remnant capable of secretome production [156].

### Re-evaluating the evidence: the importance of underlying assumptions

Research on ovarian cancer pathogenesis often rests on implicit assumptions, which can mislead interpretation. A study in 2011 reported that 100% of OICs, PTCs, and para-ovarian lesions stain for PAX8, a Mullerian tissue marker [196]. A 2018 pathologic study concluded that OICs may arise via metaplasia, based on the increasing ratio of PAX8 to calretinin (a mesothelial marker) with age relying on an assumption that ES decreases after menopause [197]. Subsequent research found that ES prevalence actually increases and persists after menopause [118], so this data showing increasing PAX8 staining with age actually supports the secretome theory, and the postulation that the increasing PAX8/calretenin ratio supports metaplasia is unwarranted.

A 2022 study which cited the above 2018 study as their reason to assume benign ovarian cysts were metaplastic presented data reported different prevalences of OIC and simple cysts in association with serous tumors and high grade carcinomas to support the ovarian metaplasia theory without reporting PAX8 staining percentages [198] making their conclusion supporting metaplasia also unwarranted. If the cystic findings reported are all considered a continuous spectrum of OICs, their data is also consistent with the uterine secretome theory, which posits a similar mechanism underlying both ubiquitous benign lesions and much less common malignant gynecologic lesions.

### Possible effect of secretome on other cancer types

Another intriguing question is whether the uterine secretome influences the development of cancers beyond the gynecologic tract. Pre-menopausal hysterectomy has consistently been associated with a reduced risk of breast cancer risk, both in prospective trials and epidemiological data [199–201]. Remarkably, only a single report of breast cancer in a MA patient who had a rudimentary uterus has been reported in the literature [202], even though the breast cancer incidence rate, 10x that of ovarian cancer, would be expected to be higher in MA patients than the general population since they are never pregnant nor breastfeed [159]. While various theories have been proposed to explain this observation, the absence of uterine-derived exosomes traveling via the bloodstream to the breast tissue to initiate ectopic growth explains this finding and warrants further investigation.

Sex-based differences in cancer incidence further support a potential systemic influence of the uterine secretome. For example, adenocarcinoma of the lungs occurs more frequently in young women compared to men [203]. Similarly, the 20-fold higher incidence of cervical cancer compared to penile cancers raises the possibility that secretome-derived factors facilitate lesion initiation in a sex-specific manner [204, 205]. These associations remain speculative but underscore the need to explore the broader impact of the uterine secretome on carcinogenesis in both reproductive and non-reproductive tissues.

## Conclusion

The “uterine secretome” theory expands upon and unifies earlier concepts, including retrograde menstruation and precursor escape, providing a biologically plausible explanation for the first step in the development of ectopic lesions, both benign and malignant. This framework resolves longstanding inconsistencies that prior theories could not explain, such as the near-ubiquitous prevalence of benign lesions in postmenopausal women, the rarity of ovarian cancer in Mullerian agenesis, and the paradoxical protective effects of tubal ligation and hysterectomy.

Further research must focus on the genetic and epigenetic drivers of implantation and their interplay with the secretome, particularly with LIF. Key questions include: which genes act in concert with LIF, how they regulate implantation pathways, and whether these pathways can be targeted to prevent ectopic lesion initiation or cancer metastasis. Although LIF has not previously been considered as the driver in the pathogenesis of ectopic growths, both LIF and its receptor are already being evaluated as a potential target for cancer treatment [206].

A deeper understanding of the role of the uterine secretome in lesion initiation will not only advance fundamental knowledge of gynecologic disease pathogenesis but also open new opportunities for early detection, refined risk assessment, and novel preventative strategies. By shifting the focus from ovulation-centered theories to endometrial secretory dynamics, the uterine secretome theory offers a new paradigm with broad implications for women’s health and cancer biology.

## Footnotes

1
https://seer.cancer.gov/statfacts/html/ovary.html
